# Synthesis and Characterization of Titanium Oxide Nanoparticles with a Novel Biogenic Process for Dental Application

**DOI:** 10.3390/nano12071078

**Published:** 2022-03-25

**Authors:** Afsheen Mansoor, Muhammad Talal Khan, Mazhar Mehmood, Zohaib Khurshid, Muhammad Ishtiaq Ali, Asif Jamal

**Affiliations:** 1 Department of Microbiology, Quaid-i-Azam University, Islamabad 45320, Pakistan; drafsheen@szabmu.edu.pk (A.M.); ishimrl@qau.edu.pk (M.I.A.); 2 Department of Dental Material Sciences, School of Dentistry, Shaheed Zulfiqar Ali Bhutto Medical University, Islamabad 44080, Pakistan; 3 Department of Dental Biomaterials, Bakhtawar Amin Medical and Dental College, Multan 60650, Pakistan; tkhaan747@gmail.com; 4 Department of Metallurgy and Materials Engineering, Pakistan Institute of Engineering and Applied Sciences, Islamabad 45650, Pakistan; mazhar@pieas.edu.pk; 5 Department of Prosthodontics and Dental Implantology, College of Dentistry, King Faisal University, Al-Hofuf 31982, Saudi Arabia; zsultan@kfu.edu.sa

**Keywords:** *Bacillus subtilis*, dental caries, glass ionomer cement (GIC), titanium oxide (TiO_2_), nanoparticles, biogenic synthesis

## Abstract

The prevalence of dental caries has been largely consonant over time despite the enhancement in dental technologies. This study aims to produce novel GIC restorative material by incorporating TiO_2_ nanoparticles synthesized by *Bacillus subtilis* for the treatment of dental caries. The TiO_2_ nanoparticles were prepared by inoculating a fresh culture of *Bacillus subtilis* into a nutrient broth for 24 h, which was then characterized by XRD, DRS, FTIR, AFM, SEM, TEM and EDX. These TiO_2_ nanoparticles were incorporated in GIC restorative material at different concentrations (0–10% TiO_2_ -GIC) and were tested for their mechanical properties in a universal testing machine. The XRD analysis revealed synthesis of anatase and rutile-phased TiO_2_ nanoparticles with a particle size of 70.17 nm that was further confirmed by SEM and TEM analysis. The EDX spectrum indicated prominent peaks of titanium and oxygen with no impurities in the prepared material. Treatment with 5% TiO_2_ -GIC proved to be most effective for the treatment of dental caries with no observable cytotoxic effect. An increase in the compressive strength of TiO_2_ nanoparticle-reinforced GIC was observed as the concentration of the TiO_2_ nanoparticles was increased up to 5%; subsequently, the compressive strength was lowered. An increase in the flexural strength was observed in GIC containing 0%, 3% and 5% TiO_2_ nanoparticles sequentially. Based on the results, it can be concluded that *Bacillus subtilis*-derived TiO_2_ nanoparticles have excellent potential for developing next generation of restorative materials for dental issues.

## 1. Introduction

The prevalence of dental caries has mainly been consonant over time despite the enhancement in dental technologies. According to World Health Organization (WHO), dental caries is considered a critical health issue for most countries in the world [1,2], affecting 3.58 billion population. The caries index has reduced in developed countries [3,4] as compared to the other nations. However, in Asia its incidence rate is still 60–90% [5] owing to poor health care systems, education and other socioeconomic factors [6]. Dental caries results from the complex interaction between fermentable carbohydrates and acid-producing tooth-adherent bacteria. Over the course of time, acid in the dental plaque initiates demineralization of the tooth structure, resulting in the onset of white spot lesion, which subsequently turns into a cavity [7]. Certain species of bacteria, including *Streptococcus mutans, Lactobacillus* spp., *Actinomyces* spp. and *Streptococcus sanguines* are involved in the initiation of dental caries, thus quoting it as a multi-microbial disease [8]. Additional factors that play a significant role in dental caries include eating habits, saliva composition, insufficient fluoride and poor oral hygiene.

Different types of restorative materials and preventive materials are used to treat dental caries, e.g., bioactive glasses, dental composites, fluoride gels and glass ionomer cements, that is referred to as GIC restorative material. Among these, GIC is the preferred restorative material because of its ability to release fluoride, its good adhesion to the tooth surface, its acceptable aesthetic effects and its excellent biocompatibility and antimicrobial properties [9,10,11]. These desirable properties of GIC restorative material have been attributed to the presence of leachable ion glass-powder and polymeric (water-soluble) acid in its formulation. On the other hand, a major drawback that diminishes the utilization of GIC restorative material in clinical practices on a large scale has been its sensitivity to moisture and compromised mechanical properties, reducing the quality of the restorative material [12,13]. There is an essential need to address these limitations of GIC restorative material in view of improving shelf-life, longevity, sustainability and durability of the contemporary tooth restoring practices. Multiple non-reactive particles, glass particles and metals have been incorporated into GIC-Matrix as a filler [14,15]. Nonetheless, achieving desired mechanical properties is a significant challenge in dentistry [16,17]. Since the start of the millennium, dendritic growth has been witnessed in nanoscience and technology [18,19]. The design principles and architecture of nanomaterials such as carbon nanotubes, nanorods and metal nanoparticles have changed the traditional outlook of material sciences. The metal-oxides nanoparticles have been tested in numerous exceptional compositions, shapes, structures, physical and chemical properties. From this perspective, titanium has become the material of choice due to its highly perceptible properties, incorporating hypoallergenicity, low toxicity, fatigue-resistance, biocompatibility [20,21], high electrical conductivity, Corrosion (Scratches) resistance, wear resisting quality [22], versatile techniques of fabrication, intrinsic properties [23,24] and cost-effectiveness [25] in comparison to the other metal-oxide nanoparticles commonly used for commercial applications [26,27].

Biogenic synthesis has been gaining a renewed interest in view of environmental benefits, cost-effectiveness and direct synthesis without consuming any additional chemicals at ambient temperature and pressure [28,29]. From this point of view, microbial synthesis has been gaining broader appreciation due to the sustainability of the product’s processing with improved properties and enhanced stability [29]. Different nanoparticles have been prepared using *Bacillus subtilis* and *Staphylococcus aureus*. Besides extensive research on the microbial synthesis of nanoparticles, knowledge and understanding are scarce regarding the mechanism of biosynthesis, size and shape control, particularly in metal oxide nanoparticles [29,30]. Previously, the mechanical properties of GIC restorative materials have been improved by the application of commercially available TiO_2_ nanoparticles in a uniform anatase phase with a particle size of 25 nm [31,32]. It is worth mentioning that anatase phase and reduced size have more excellent surface absorption to impose cytotoxic effects of the prepared nanomaterials, which therefore restricts their implications in the restorative formulations, despite showing excellent mechanical properties [33,34,35,36]. 

Previous studies have suggested that biogenically synthesized metal-based nanoparticles demonstrate antiseptic effects, reduce infections in wounds and promote healing of wounds [37]. Another study carried out on biogenically synthesized TiO_2_ nanoparticles depicted that these nanoparticles possessed antibacterial properties [38]. The antimicrobial effects of the TiO_2_ nanoparticles prepared in this study will be evaluated and discussed in future studies

In the present research, TiO_2_ nanoparticles were synthesized by *Bacillus subtilis* (a nonpathogenic, gram-positive bacteria) and then characterized by SEM, TEM, AFM, EDX, FTIR, XRD and DRS. This novel GIC restorative material containing TiO_2_ nanoparticles was tested for its mechanical strength and surface morphology to treat dental caries, to ensure enhanced biocompatibility, shelf life and durability as an improved dental restorative material.

## 2. Materials and Methods

### 2.1. Microbial Synthesis of TiO_2_ Nanoparticles

Bacterial culture in the present work was prepared using *bacillus subtilis* (Accession No: ATCC^®^6633^TM^, Catalog No: 0486SPR), which was taken from the National Institute of Health (NIH), Islamabad, Pakistan. The microbial synthesis of TiO_2_ nanoparticles was carried out according to the methodology mentioned in the previously published literature [39]. 

Synthesis of TiO_2_ nanoparticles was performed by inoculating the fresh culture of *Bacillus* *subtilis* (Accession No: 93 ATCC^®^6633^TM^, Catalog No: 0486SPR) into 100 mL of nutrient broth in Erlenmeyer flask and incubated for the next 24 h by placing them in a shaking incubator (Memmert, Schwabach, Germany) at 28 °C and 150 rpm. Subsequently, all the content of the flask was subjected to centrifugation at 10,000 rpm for 10 mins in order to obtain the cell-free filtrates. The supernatant was separated and 20 mL of commercial 0.025 M Ti(OH)_2_ solution (American Elements, 10,884 Weyburn Ave. Los Angeles, CA90024, USA) was added to 80 mL bacterial culture solution and heated on a steam bath at 60 °C for 10–20 min until white deposition started to appear at the bottom of the flask. After 12–48 h, white deposition in the solution took the form of nanoparticles when kept in laboratory ambience. Finally, TiO_2_ nanoparticles were centrifuged (centrifuge model; MOD-800) and then annealed in the furnace (Thermo Fisher Scientific, Waltham, MA, USA) at 80 °C for 2 h to obtain entirely dried TiO_2_ nanoparticles. The TiO_2_ nanoparticles obtained were eventually calcinated in a furnace (Thermo Fisher Scientific, Waltham, MA, USA) at 500 °C for 3 h to attain a fine powdered form of TiO_2_ nanoparticles [39].

### 2.2. Characterization of TiO_2_

The D/MAX-2400 diffractometer (Rigaku Corporation, Akishima Tokyo, Japan, λ = 0.154181 nm) was used to identify different crystalline structures and phases of TiO_2_ nanoparticles. A UV/VIS/NIR Spectrometer (Lambda 950, Perkin Elmer, Waltham, MA, USA), was utilized to reveal the TiO_2_ nanoparticles’ energy structures and optical properties. The surface morphology in terms of roughness of TiO_2_ samples was studied using Atomic Force Microscopy (Quesant Universal SPM, Ambios Technology, Santa Cruz, CA, USA). A tapping-mode AFM probe with a cantilever tip, HQ: NSC-16 (Mikromasch), was used. Scanning electron microscopy (NOVA NanosemNO: 430, FEI- company, Hillsboro, OR, USA) and Transmission Electron Microscopy (Jeol JEM-200CX, Bioz Stars, Tokoyo, Japan) were deployed for characterizing the surface morphology of TiO_2_ nanoparticles. Qualitative element analysis of specimens was performed by energy-dispersive x-ray spectroscopy by utilizing a spectrophotometer (NOVA Nanosem 430, FEI-company, Hillsboro, OR, USA). The Fourier Transform Infrared spectrophotometer (JASCO FT/IR-6600, Utrecht, the Netherlands) was employed to determine functional groups of TiO_2_ [40].

### 2.3. Cytotoxicity Testing for Biocompatibility of TiO_2_ Nanoparticles

#### 2.3.1. MTT Assay

The cytotoxicity analysis of TiO_2_ nanoparticles was performed using L929 mouse fibroblasts (ATCC, Manassas, VA, USA) through MTT assay. These fibroblasts were maintained in standard culture conditions [41]. The temperature was maintained at 37 °C in 5% CO_2_ at 95% humidity. Subsequently, trypsinization was performed, and the cell suspension was made in 10% DMEM containing 1 × 10^4^ cells [42]. Thereafter, 100 µL of cell suspension was obtained and seeded in each well of the standard 96-well plate for 24–48 h. The TiO_2_ nanoparticles were added as 1 mg/mL stock solution concentration to test cytotoxicity. MTT dye (Sigma Aldrich, Saint Louis, MO, USA) was added to each well, and the plates were incubated at 37 °C for 2 h. The fluorescence of each well was measured at a wavelength of 490 nm with a fluorescence well plate reader (Thermo Fisher, Waltham, MA, USA) at day-1, day-3, day-5, day-7, day-21 and day-30 [43].

#### 2.3.2. Morphology

Morphology was investigated by taking the images of cells in a 96-well plate with an Inverted Fluorescence Microscope (Euromex, Arnhem, the Netherlands) [41].

### 2.4. Preparation of TiO_2_-GIC Samples for Flexural and Compressive Strength Testing

A total of 100 samples (*n* = 10) belonging to different experimental groups having different concentrations of TiO_2_ nanoparticles were incorporated in GIC restorative material to make TiO_2_-GIC samples and to find out what concentration of these samples was strong enough to enhance the mechanical properties of this novel GIC restorative material.

The different concentrations of these TiO_2_ nanoparticles incorporated in the GIC were: control group = 1 (0 wt% TiO_2_-GIC), experimental Group = 2 (3 wt% TiO_2_-GIC), experimental Group = 3 (5 wt% TiO_2_-GIC) experimental Group = 4 (7 wt% TiO_2_-GIC), experimental Group = 5 (10 wt% TiO-GIC).

#### 2.4.1. Flexural and Compressive Strength Testing

Samples for flexural strength testing (*n* = 50) and compressive strength testing (*n* = 50) for all different concentrations of TiO_2_-GIC powder samples were prepared according to previously published research. The three point bending in the Universal Testing Machine (Shenzhen- SANS, Testing-Machine, Co-Ltd., Nanshan, Shenzhen, China) was performed to test the flexural strength by placing samples in a cylinder with an opening of about 10 mm in diameter and a load at a cross speed of 1 mm/min mpa was employed. The TiO_2_-GIC samples were divided into two parts and measurements were obtained. Moreover, two flat metal disks in the Universal Testing Machine (Shenzhen-SANS, Testing-Machine Co-Ltd., Nanshan, Shenzhen, China) were used for compressive strength testing of the prepared TiO_2_-GIC samples. These TiO_2_-GIC samples were subjected to a compressive load at a cross speed of about 1 mm/min mpa until the TiO_2_-GIC samples fractured. This procedure was repeated for all samples and results were obtained.

#### 2.4.2. Scanning Electron Microscope Analysis:

Standardized TiO_2_-GIC sample blocks for all different concentrations were polished with different silicon carbide abrasive papers in a metallographic polishing machine (Scientific, Instrument Measurement and Control Co-Ltd., Nanjing, China). These TiO_2_-GIC sample blocks were sputter-coated in a Sputter Coating Machine (Quorum: Technologies Ltd., Lewes, UK) for at least 30 min [44]. The cross-section of these sample blocks was investigated under SEM (Nova-Nanosem, NO: 430-FEI-Company, Hillsboro, OR, USA).

## 3. Results

### 3.1. Color Changes after Synthesis

The synthesis of the nanoparticles was carried out using supernatant of *Bacillus subtilis* in shake flask experiments. The color of bacterial substrate solution changed from yellowish-cream to white, indicating the formation of TiO_2_ nanoparticles after 20 min.

### 3.2. Characterization

#### 3.2.1. X-ray Diffraction Analysis (XRD)

**Figure 1:**
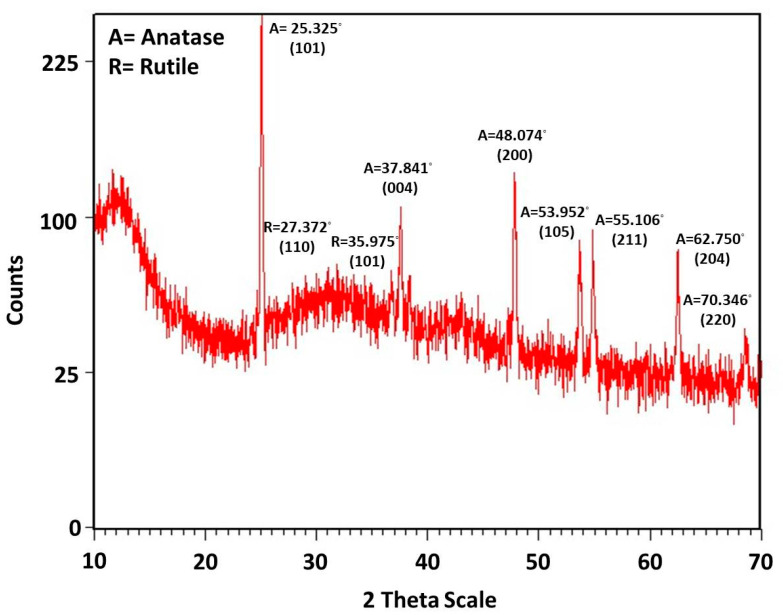
XRD pattern of TiO_2_ nanoparticles synthesized by *Bacillus subtilis*.

X-ray diffraction analysis of the TiO_2_ nanoparticles was carried out to ascertain the crystallite size of the particles and to identify the crystalline phases present. The size of the crystals was determined by Scherrer’s equation (Equation (1)):

τ(D) = Kλ/βcos(θ)(1)

where D = average crystallite size, K = shape factor “0.9”, λ = wavelength of X-ray 1.5406 Å Cu- K_α_ radiation and θ = Bragg angle and β = line broadening at half the maximum intensity (FWHM). X-ray diffraction analysis pattern of TiO_2_ nanoparticles synthesized by *Bacillus subtilis* was found to give a mixed anatase and rutile phases. The peaks matched well with the Joint Committee on Powder Diffraction Standards (JCPDS card no: 01-084-1286) with the main peak [101] of anatase phase at 2θ = 25.325°. Several other peaks were observed at [004] = 37.84°, [200] = 48.07°, [105] = 53.95°, [211] = 55.11°, [204] = 62.75° and [220] = 70.346°. The peaks related to the rutile phase were (JCPDS card no: 01-077-0442) observed at 2Ѳ [110] = 27.372 and [101] = 35.975. The crystalline size of TiO_2_ nanoparticles synthesized with *Bacillus subtilis* was calculated by the Debye–Scherrer’s formula, which was found to be 70.00 nm (Figure 1). The nanoparticles synthesized consisted of 52% pure anatase phase and 48% pure rutile phase.

#### 3.2.2. Diffuse Reflectance Spectroscopy (DRS)

**Figure 2:**
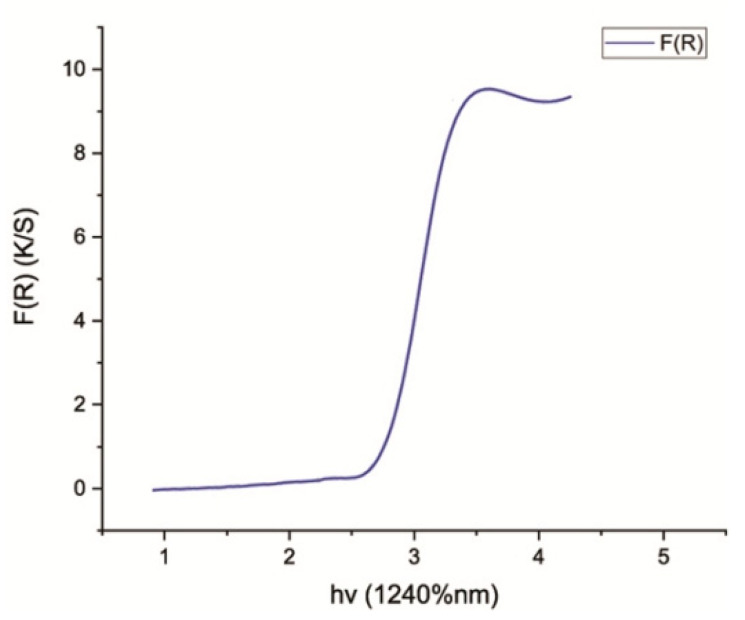
DRS pattern, showing energy band gap of TiO_2_ nanoparticles synthesized by *Bacillus subtilis*.

The Eg (band gap energy) for pure TiO_2_ nanoparticles synthesized by *Bacillus subtilis* was calculated from wavelength values corresponding to the intersection points of horizontal and vertical areas in the spectrum plotted from UV-Vis spectroscopy data. The shift of reflectance spectrum from higher wavelength to lower wavelength elaborated the crystallite size of TiO_2_ nanoparticles. The standard value for Eg was 3.23 eV. The values of Eg verified the crystallite size/particle size of TiO_2_ nanoparticles. When the calculated value of Eg is greater than its standard value (3.23 eV), the crystallite size/particle size of the synthesized nanoparticles decreases. On the other hand, when the calculated Eg is less than 3.23 eV, the crystallite size/particle size of the synthesized nanoparticles increases. This shows that an inverse relationship exists between Eg and crystallite size/particle size of nanoparticles [45]. Therefore, the band gap energy calculated for TiO_2_ nanoparticles synthesized by *Bacillus subtilis* was found to be 2.8 eV, confirming that the crystallite size of these TiO_2_ nanoparticles was larger (Figure 2).

#### 3.2.3. Atomic Force Microscopy (AFM)

**Figure 3:**
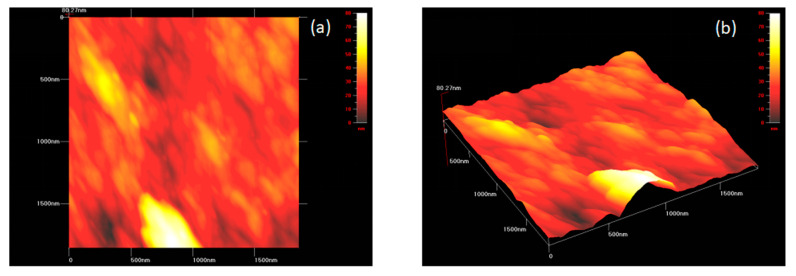
A three-dimensional image of TiO_2_ nanoparticles synthesized by *Bacillus subtilis* obtained using atomic force microscope at (**a**) low resolution and (**b**) high resolution.

Atomic force microscopy was carried out to observe the three-dimensional topography of the TiO_2_ nanoparticles synthesized by *Bacillus subtilis.* The surface morphology of TiO_2_ nanoparticles revealed an uneven and bumpy surface with minimal roughness, respectively. This became possible as a result of the availability of individual as well as accumulated aggregates of TiO_2_ nanoparticles (Figure 3a,b). 

#### 3.2.4. Scanning Electron Microscope (SEM) 

**Figure 4:**
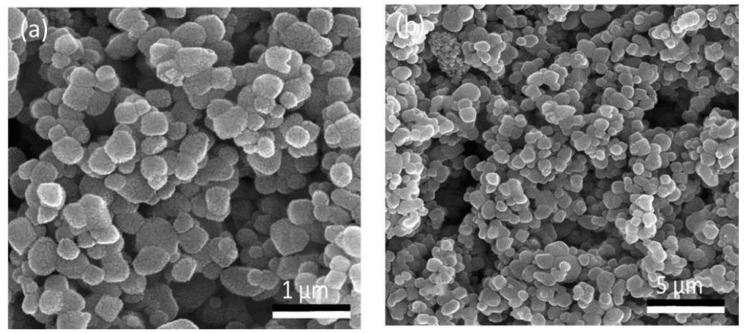
Scanning electron microscopic image of TiO_2_ nanoparticles at (**a**) 1000× (**b**) 5000×.

Scanning electron microscopy of the TiO_2_ nanoparticles revealed predominantly spherical-shaped clusters with an adequate dispersion at 1000× (Figure 4a). Furthermore, these nanoparticles exhibited a regular smooth structure with evenly distributed particles at a higher magnification of 5000× (Figure 4b). In addition, the structure of the particles displayed a mixture of dominantly spherical-shaped particles with a few oval-shaped particles in it that were found individually and in aggregates with a particle size of 70.17 nm (Figure 4).

#### 3.2.5. Transmission Electron Microscope (TEM)

**Figure 5:**
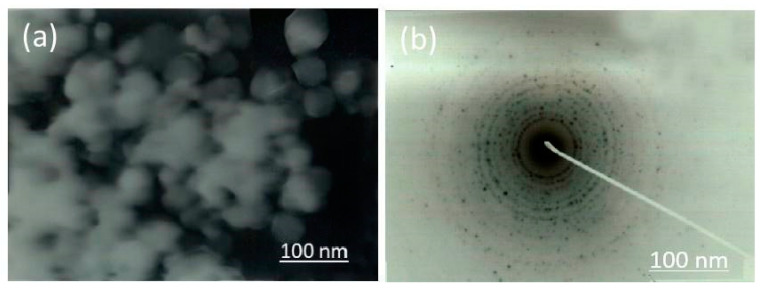
Transmission electron microscopic image of (**a**) TiO_2_ nanoparticles synthesized by *Bacillus subtilis* (**b**) Selected area electron diffraction peaks.

Transmission electron microscopy (TEM) elucidated that the TiO_2_ nanoparticles were finely spherical-shaped and were present both individually and in aggregates. The size of the nanoparticles was around 70.17 nm (Figure 5a). Therefore, the Selected Area Electron Diffraction (SAED) image expressed the crystallinity acquired by these nanoparticles (Figure 5b). The results obtained were in collaboration with XRD and SEM analysis.

#### 3.2.6. Energy Dispersive X-ray Analysis (EDX)

**Figure 6:**
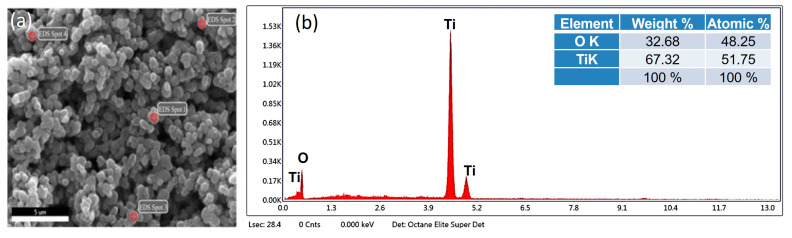
Energy dispersive X-ray spectroscopic analysis of TiO_2_ nanoparticles displaying the (**a**) SEM image with EDX Spots. (**b**) Elemental composition showing peaks of titanium and oxygen.

The energy dispersive X-ray spectroscopic analysis of the nanoparticles revealed an intense peak of titanium (Ti) and oxygen (O) in its spectrum. The weight percentage (wt%) and atomic percentage (%) of titanium (Ti) in the EDS spectrum were 69.71% and 50.05%, respectively, whereas the weight percentage (wt%) and atomic percentage (%) of oxygen (O_2_) were 30.29% and 49.95%, respectively (Figure 6).

#### 3.2.7. Fourier Transmission Infrared Spectroscopy (FTIR)

**Figure 7:**
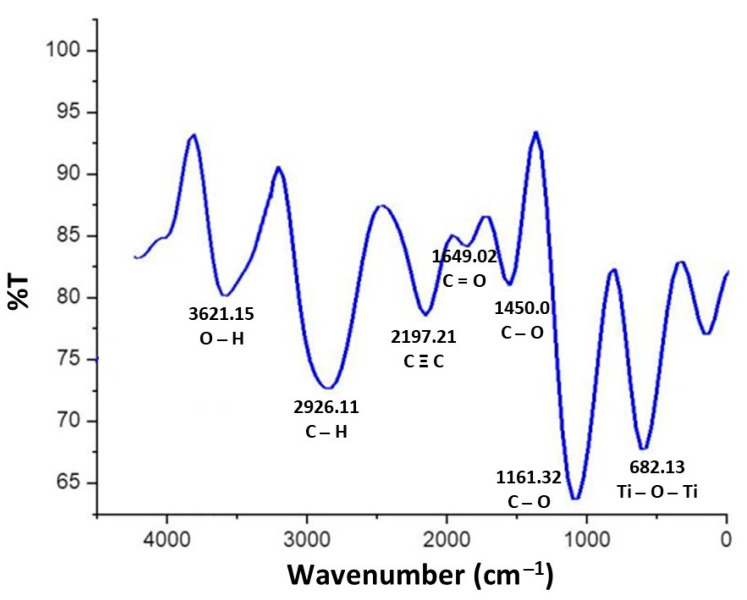
FTIR spectrum of TiO_2_ nanoparticles synthesized by *Bacillus subtilis*.

The FTIR spectrum of the TiO_2_ nanoparticles revealed peaks at 3621.15 cm^−1^, 2926.11 cm^−1^, 2197.21 cm^−1^, 1649.02 cm^−1^, 1450.07 cm^−1^, 1161.32 cm^−1^ and 682.13 cm^−1^. The peak at 3621.15 cm^−1^ revealed O–H stretching due to the presence of alcohol in its composition. The C–H symmetric, non-symmetric C=O stretching and C–O stretching frequencies were observed at 2926.11 cm^−1^, 1649.02 cm^−1^ and 1161.32 cm^−1^, respectively. Thus, the peak at 2926.11 cm^−1^ corresponded to the carboxylic group, whereas the peaks of 1649.02 cm^−1^, 1450.07 cm^−1^ and 1161.32 cm^−1^ confirmed the presence of primary as well as secondary amines and their linkages. The prominent peak of Ti–O–Ti bending at 682.13 cm^−1^ exhibited the presence of metal oxygen bonds, revealing the formation of TiO_2_ nanoparticles. This is due to the reason that presence of Ti–O–Ti bending peak might be responsible for the formation of TiO_2_ nanoparticles (Figure 7).

### 3.3. Biocompatibility Investigation of TiO_2_ Nanoparticles

#### 3.3.1. MTT Assay

MTT assay was employed to assess the cytotoxicity of TiO_2_ nanoparticles. For the purpose of assessing cytotoxicity, an extract (*n* = 5) was prepared from TiO_2_ nanoparticles and a comparison with control group was established. The control group had a cell survival rate of 100% at days 1, 3, 7, 21 and 30. The extract from nanoparticles exhibiting a cell survival rate of greater than 90% in comparison to the control group were considered non-cytotoxic, while the extract from those exhibiting a cell survival rate between 60–90% were considered mildly cytotoxic. The extract from nanoparticles showing cell survival rates of 30–60% were considered moderately cytotoxic, while the extract from the nanoparticles with a cell survival rate lower than 30% were considered severely cytotoxic. The cell survival rate of each of the TiO_2_ nanoparticles was calculated via the following formula:

(2) $$\mathrm{Cell}\;\mathrm{viability}\;\%=\frac{\mathrm{Mean}\;\mathrm{optical}\;\mathrm{density}\;\mathrm{of}\;\mathrm{test}\;\mathrm{group}}{\mathrm{Mean}\;\mathrm{optical}\;\mathrm{density}\;\mathrm{of}\;\mathrm{control}\;\mathrm{group}}\times 100\%$$

#### 3.3.2. Cell Viability

**Figure 8:**
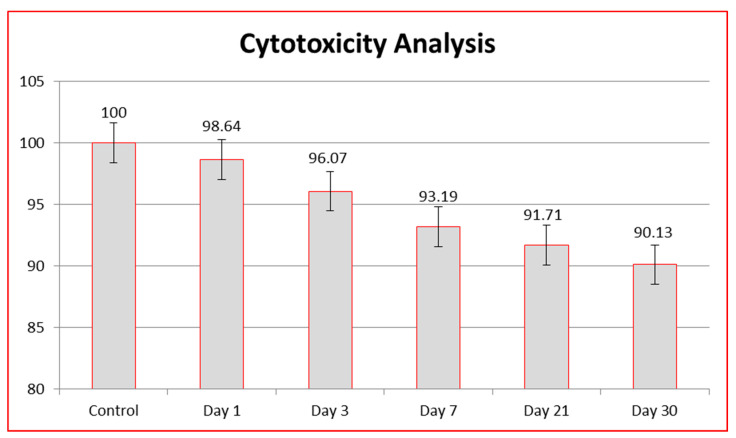
Cell viability (%) of TiO_2_ nanoparticles at various days in comparison to the control group.

The cytotoxicity of TiO_2_ nanoparticles was calculated in terms of cell viability at different days within 1 month duration. The cell viability values closer to the control group means greater then 90% showed the non-cytotoxic behavior of the synthesized nanoparticles, while cell viability values less than 90% in comparison to the control group (water) revealed the cytotoxic behavior of the TiO_2_ nanoparticles. The cell viability relating cytotoxicity was recorded at day 1, day 3, day 7, day 21 and day 30. The cell viability of the control group was 100%. During the first 24 h, the TiO_2_ nanoparticles had the highest cell viability (cell viability = 98.64%). This was followed by cell viability at day 3 (cell viability = 96.07%) and day 7 (cell viability = 93.19%). The lowest cell viability was observed at day 21 (cell viability = 91.71%) and day 30 (cell viability = 90.13%). The cell viability was lower than the control group on each day and was statistically significant (*p* < 0.001), which meant that the cell viability of the synthesized TiO_2_ nanoparticles reduced with each passing day as compared to the control group, which was 100% at each day of analysis. However, the cell viability of TiO_2_ nanoparticles was significantly less than the control group (100%) but fell within the range of non-cytotoxicity. The comparison of cell viability to the control group has been provided (Table 1, Figure 8). Thus, TiO_2_ nanoparticles were found to be non-cytotoxic from day 1 to day 30 (cell viability > 90) (Figure 8). The cytotoxicity of TiO_2_ nanoparticles were compared with each other at different days to find out the effect of duration on the development of cytotoxicity. This comparison displayed that the reduction in cell viability on day 30 was more as compared to the day 1, but still the values reported were greater than 90%, which shows their non-cytotoxic behavior. Hence, comparison of cytotoxicity of TiO_2_ nanoparticles at day 1, 3, 7, 21 and 30 was significant but within non-cytotoxic range (*p* < 0.00) (Table 2).

#### 3.3.3. Morphology 

**Figure 9:**
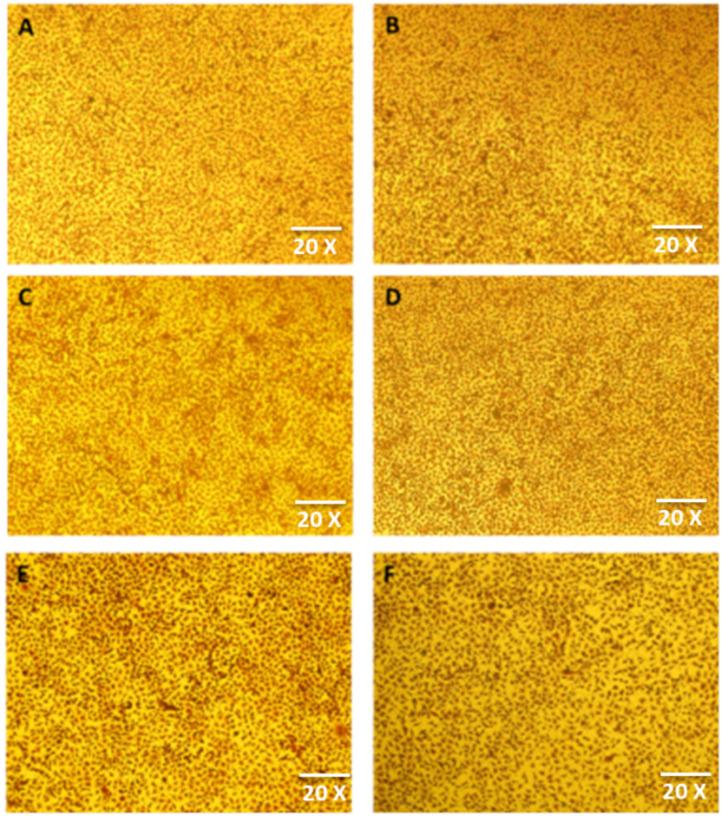
Morphology of (**A**) cells in control group at day 1, (**B**) TiO_2_ nanoparticle-treated cells at day 1, (**C**) cells in control group at day 7, (**D**) TiO_2_ nanoparticle-treated cells at day 7, (**E**) cells in control group at day 30, and (**F**) TiO_2_ nanoparticle-treated cells at day 30.

The L929 fibroblasts used in this study were spindle-shaped with processes extending out from the cells. The fibroblasts had a branched cytoplasm that surrounded an elliptical and speckled nucleus with two or more nuclei. The cytoplasm had abundant bundles of rough endoplasmic reticulum and large Golgi apparatus. No change in morphology of the cells was observed at the end of day 1 and 30 in the control group and in cells exposed to the extract of TiO_2_ nanoparticles (Figure 9A–D).

### 3.4. Mechanical Strength Testing of TiO_2_ GIC

#### 3.4.1. Compressive Strength Analysis

**Figure 10:**
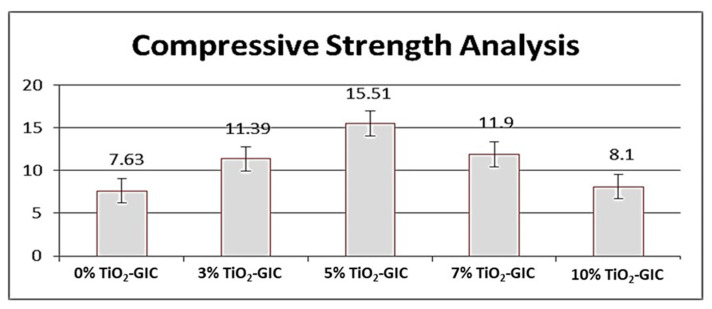
Compressive strength analysis of GIC restorative material containing various concentrations of TiO_2_ nanoparticles.

An increase in the compressive strength of TiO_2_ nanoparticles reinforced in GIC restorative material was observed as the concentration of the TiO_2_ nanoparticles was increased up to 5%; subsequently, the compressive strength was lowered with the further addition of TiO_2_ nanoparticles in GIC restorative material. The highest compressive strength was observed in 5% TiO_2_-GIC samples and lowest compressive strength was observed in 10% TiO_2_-GIC samples as compared to control group 0% TiO_2_-GIC samples (Figure 10) The compressive strength of inter groups with different concentration of TiO_2_ nanoparticles (TiO_2_-GIC samples) was also calculated to find out the mean difference in the compressive strength among these samples. The intergroup comparisons of compressive strength of GIC restorative material containing various concentrations of TiO_2_ nanoparticles have been shown in (Table 3).

#### 3.4.2. Flexural Strength Analysis

**Figure 11:**
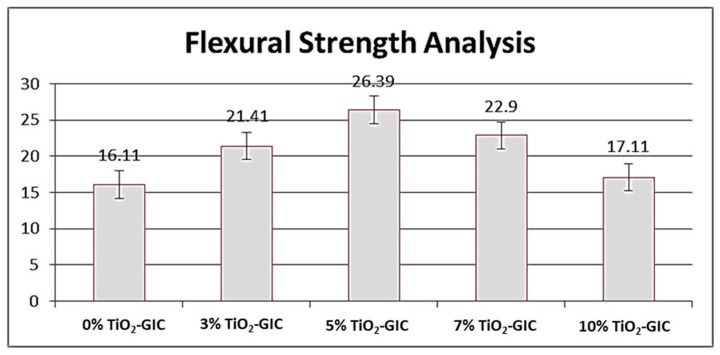
Flexural strength analysis of GIC restorative material containing various concentrations of TiO_2_ nanoparticles.

TiO_2_ nanoparticles synthesized by *Bacillus subtilis* were added to GIC restorative material in various concentrations and flexural strength was measured. An increase in the flexural strength was observed in GIC restorative material containing 0%, 3% and 5% TiO_2_ nanoparticles sequentially (Figure 11). However, a decrease in flexural strength was observed in GIC restorative material containing 7% and 10% TiO_2_ nanoparticles. Nevertheless, the highest flexural strength was recorded in 5% TiO_2_-GIC samples, whereas the lowest flexural strength was observed in 10% TiO_2_-GIC samples as compared to the control group 0% TiO_2_-GIC samples containing no TiO_2_ nanoparticles. The intergroup comparisons of flexural strength of GIC restorative material containing various concentrations of TiO_2_ nanoparticles have been shown in (Table 4).

#### 3.4.3. Scanning Electron Microscopy (SEM)

**Figure 12:**
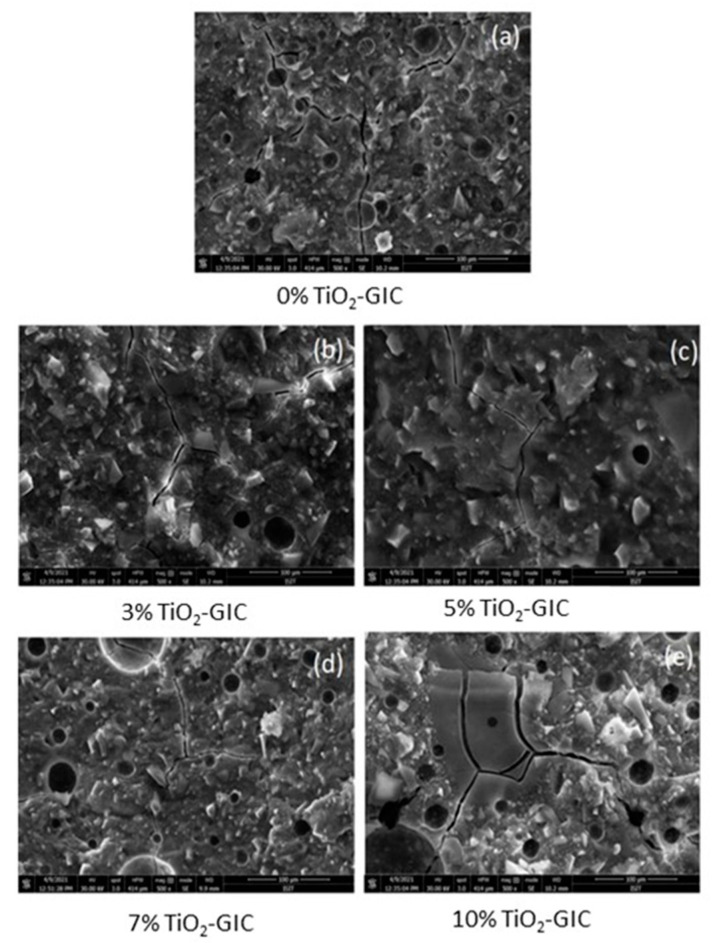
SEM images of TiO_2_-GIC samples at different concentrations after flexural strength testing ((**a**) = 0%TiO_2_-GIC, (**b**) = 3% TiO_2_-GIC, (**c**) = 5% TiO_2_-GIC, (**d**) = 7% TiO_2_-GIC and (**e**) = 10% TiO_2_-GIC).

Scanning electron microscopy analysis of GIC restorative material samples containing 0%, 3%, 5%, 7% and 10% TiO_2_ nanoparticles were assessed. The structure of 0% TiO_2_-GIC revealed a highly porous structure and micro-cracks (Figure 12a). The 3% TiO_2_-GIC samples and 5% TiO_2_-GIC samples (Figure 12b,c) exhibited lower porosity and micro-cracks as compared to the 7% TiO_2_-GIC samples and 10% TiO_2_-GIC samples, which revealed higher porosity and micro-cracks (Figure 12d,e). Thus, 5% TiO_2_-GIC samples (Figure 12c) depicted lowest porosity and micro-cracks as compared to 10% TiO_2_-GIC samples, which revealed the highest porosity and micro-cracks (Figure 12e).

## 4. Discussion

The TiO_2_ nanoparticles were prepared through a biogenic route in the current study with the help of *Bacillus subtilis*, which were then characterized and checked for their biocompatibility. These nanoparticles were incorporated into GIC restorative material to produce a novel, bio-safe and biocompatible TiO_2_-GIC restorative material that exhibited improved mechanical properties to bear masticatory loads in the oral cavity. These nanoparticles were prepared without any artificially added capping agent, reducing materials, templates or toxic chemicals. Moreover, lower temperature, pressure and energy was employed in the preparation. Thus, nanoparticles synthesized in this study possessed a higher biocompatibility as compared to those prepared by conventional techniques. It has been reported that nanoparticles prepared with the help of microbial molecules have a higher precision, size and shape control [46,47]. These nanoparticles are more stable because of their quick formation at low temperature, pressure and pH, which simultaneously makes them cost-effective [48]. The change of color is a key indication and confirmation of synthesis of nanoparticles [49]. The mechanism responsible for color change of TiO_2_ nanoparticles could be the oxidation of metallic Ti originating from precursor salts, metal ions of Ti breaks into Ti^2+^ cations and Ti^2−^ anions by reacting with water molecules present in these precursor salts. The electron is released that might have been taken up by Ti^2+^ cations along with water resulting in change of color from dark to light. The XRD analysis indicated that (Figure 1) the reaction produced a mixture of 52% pure anatase phase and 48% pure rutile phase with a particle size of about 70.17 nm. This is possible due to the reason that heating in the oven first and then in the furnace might have produced mixture of anatase and rutile phase of TiO_2_ nanoparticles. These phases are very important in terms of practical implications of these nanoparticles. The band gap energy of TiO_2_ nanoparticles revealed by DRS (Figure 2) was found to be 2.8 eV as compared to the standard value of 3.32 eV, supporting the fact that smaller crystallite/particle size constituted larger energy band gap and vice versa [45,50]. This confirmed that crystallite/particle size of these biogenically synthesized TiO_2_ nanoparticles was larger. The presence of a large number of biomolecules, such as enzymes, proteins and co-enzymes, in bacteria might have activated the secondary reduction process. This reaction increases the adsorption of metal-ions on preformed-nuclei surfaces in turn, leading to the formation of large-sized nanoparticles [51]. The AFM analysis (Figure 3) displayed a smooth surface of nanoparticles due to the accumulation of a large amount of TiO_2_ nanoparticles on the surfaces [52,53]. The smooth nature of synthesized TiO_2_ nanoparticles contributed towards the uniform capping of their surfaces during the synthesis. The plausible explanation for this uniform cap formation on the surfaces of TiO_2_ nanoparticles could be the result of enzymes released by *Bacillus subtilis*, which gave rise to the particularly large size and spherical shape of these nanoparticles.

Scanning electron microscopy and transmission electron microscopy (Figure 4 and Figure 5) depicted a spherical shape with a particle size of about ~70.00 nm in diameter [54]. Factors responsible for such s size, shape and surface topography of TiO_2_ nanoparticles are pH, time, temperature and the reducing agents involved in the reaction [55]. There are two possible explanations for generating nanoparticles of variable sizes and shapes. Firstly, the availability of large amounts of natural reducing agents and precursors might accelerate the secondary reduction of metallic ions on nuclei’s surfaces after initial bonding of preformed nuclei on the surface of metal ions resulting in spherical large sized nanoparticles. Secondly, the presence of increased amounts of natural reducing agents and precursors might enhance bridging among nanoparticles, leading to aggregation of nanoparticles by enhancing secondary reduction of metallic ions [51].

The EDX spectroscopy (Figure 6) made clear the presence of titanium (Ti) and oxygen (O) peaks without any impurities in nanoparticles that might result in undesirable properties including cytotoxicity. The components used as reducing and stabilizing agents in biogenic synthesis are present inside microorganisms as enzymes, proteins and co-enzymes [56]. These nanoparticles are considered as biologically safe, ecofriendly and non-toxic [57,58,59], thus confirming a high biocompatibility of these biogenically synthesized TiO_2_ nanoparticles for use in biomaterials. The FTIR spectroscopy (Figure 7) revealed the presence of characteristic Ti–O–Ti stretching band peaks between 800 cm^−1^–400 cm^−1^. Similarly, peaks observed between 600–400 cm^−1^ demonstrated a bending vibration of Ti–O–Ti bonds. These Ti–O–Ti stretching and Ti–O–Ti bending peaks in these assigned wave lengths are basically responsible for the production of the TiO_2_ nanoparticles [60]. The presence of bacterial proteins and lipids play a key role in producing amine linkages that are essentially beneficial in microbial synthesis of nanoparticles [52]. These amine linkages help in the nucleation of TiO_2_ nanoparticles by helping proteins to bind with metallic nanoparticles [52]. Thus, the involvement of only bacterial proteins and lipids during synthesis play a key role in defining the characteristics of these nanoparticles.

The cytotoxicity analysis of TiO_2_ nanoparticles demonstrated non-cytotoxic behavior because of their cell viability >90% at all the days. TiO_2_ nanoparticles synthesized in this study were prepared through a biogenic route with the help of *Bacillus subtilis* that justifies the higher biocompatibility of these nanoparticles. The analysis of cell morphology (Figure 9) at the end of day 30 revealed no changes when compared to the control group. The cytotoxicity of nanoparticles is predominantly dependent on their mode of synthesis, physico-chemical properties, time-duration of exposure and concentration. Previously, nanoparticles became cytotoxic in nature because of utilization of different toxic and expensive chemicals in their synthesis [57,58,59]. Additionally, the nanoparticles prepared by conventional methods were also found to be unstable, to have produced hazardous byproducts and to have attached toxic substances on their surfaces. The size, shape, phases and surface area topography of nanoparticles plays a great role in developing cytotoxicity because there exists a direct relationship between physico-chemical properties of synthesized nanoparticles and cytotoxicity [61]. In addition, an inverse relationship occurs between physico-chemical properties (size and shape) and surface area to volume ratio of nanoparticles. If the nanoparticles are smaller and irregular in shape, they have larger surface area to volume ratio, which means that they are easily and quickly adsorbed in any surface or cell lines, giving them greater cytoxicity. Similarly, if nanoparticles are larger in size and spherical in shape, they have a lesser surface area to volume ratio, which means that they are arduously and slowly adsorbed in any surface or cell lines, making them non-cytotoxic. This justifies the non-cytotoxic behavior of TiO_2_ nanoparticles synthesized in the current study. Thus, these nanoparticles became capable of inhibiting the production of reactive oxygen-species (ROS) and free-radicals, which are solely responsible for producing cytotoxicity.

The mixture of anatase and rutile phases of TiO_2_ nanoparticles produced in the current study are least reactive and more stable because of their high surface energy, which makes them more biocompatible and noncytotoxic. Moreover, it has been reported that the anatase phase is considered as more reactive and unstable in addition to contributing to the toxicity in the product. This is possibly due to the low surface energy of anatase phase as compared to brookite and rutile phases [62,63]. This low surface energy of anatase phase might be responsible for attacking cell organelles more quickly and causing cytotoxicity, as compared to the mixture of anatase–rutile phases. The presence of smooth layer formation in current study as a result of high concentration of TiO_2_ nanoparticles on the surface makes them biocompatible. The irregular surfaces may result in microcracks, accelerated degradation and the release of constituents, which may contribute to the cytotoxicity of a material as it provides a higher surface area to volume ratio.

The restorative materials used in oral cavity must be strong enough to bear masticatory loads applied on them. The compressive and flexural strength are very important for bearing excessive masticatory loads [64]. The *Bacillus subtilis*-mediated TiO_2_ nanoparticles were incorporated in GIC restorative material as a result of its highest level of biocompatibility. The novel TiO_2_-GIC restorative material was tested at different concentrations of TiO_2_ nanoparticles to identify the ideal concentration for restoration. The 5% TiO_2_-GIC samples revealed maximum compressive (15.51 ± 0.39) and flexural strength (26.39 ± 0.43), while 10% TiO_2_-GIC samples displayed a minimum compressive (8.10 ± 0.37) and flexural strength (17.11 ± 0.24) when compared to control group containing 0% TiO_2_-GIC samples without TiO_2_ nanoparticles. Surface morphology revealed minimum voids with cracks in 5% TiO_2_-GIC samples (Figure 12c) as compared to 0% TiO_2_-GIC samples and 10% TiO_2_-GIC samples (Figure 12a,e) containing maximum voids with cracks.

The GIC restorative material without TiO_2_ nanoparticles contain voids in its structure due to entrapment of air bubbles, thus reducing its compressive strength [14,65]. The incorporation of TiO_2_ nanoparticles into GIC restorative material reduced and completely filled the spaces between the particles of GIC restorative material. This, in turn, enhanced the consistency and homogeneity of the modified version of TiO_2_-GIC restorative material, resulting in an increased compressive strength [66]. The reason for maximum increase in novel 5% TiO_2_-GIC restorative material might be due to the availability of an adequate quantity of GIC restorative material’s particles to bind with TiO_2_ nanoparticles, thus reducing any chance of the presence of free and unbound TiO_2_ nanoparticles [65]. Similarly, an added percentage of TiO_2_ nanoparticles into GIC restorative material will, to a certain extent, promote the release of metal ions along with the formation of cross-linking agents, thereby resulting in increased compressive strength [67]. Additionally, excessively increased percentage of added TiO_2_ nanoparticles will prohibit the release of metal ions and will prevent formation of cross-linking agents, thereby reducing compressive strength [66,68].

The increase in flexural strength is closely associated with integrated micro-structure [69]. The particle size of TiO_2_ nanoparticles and cross linkings between them at the mixing stage are important factors responsible for enhancing flexural strength of GIC restorative material. A plausible explanation could be that TiO_2_ nanoparticles are available in nano-scale; therefore, they occupy all large empty gaps present in GIC restorative material to a certain limit [70]. The more TiO_2_ nanoparticles that are added, the more their number and surface area increases. At this point, the amount of GIC restorative material’s particles are insufficient to bind with TiO_2_ nanoparticles. This, in turn, will result in an increased number of free and unbound TiO_2_ nanoparticles in GIC restorative material, which will reduce its flexural strength [71].

## 5. Conclusions

The current study concluded that biogenically synthesized TiO_2_ nanoparticles utilizing *Bacillus subtilis* were biocompatible and non-cytotoxic in nature because of their higher precision, stability, size and shape control. Therefore, these TiO_2_ nanoparticles were potential filler materials when employed in GIC restorative material used as restorative material in dentistry. The novel GIC restorative material containing TiO_2_ nanoparticles at 5% concentration (TiO_2_-GIC) displayed improved mechanical strength and specifically compressive and flexural strength to treat dental caries with enhanced biocompatibility, shelf life and durability of this novel restorative material.

## 6. Limitations and Future Considerations

The metallic nanoparticles are well known for their potent antimicrobial activity. Therefore, further analyses regarding antimicrobial activity of biogenically synthesized TiO_2_ nanoparticles are recommended. The current study reported the flexural strength and compressive strength of GIC restorative material incorporated with different concentrations of TiO_2_ nanoparticles. The shear bond strength of different concentrations of TiO_2_-GIC restorative materials in accordance with enamel and dentine should also be calculated. 

## Figures and Tables

**Table 1:** Comparison of cytotoxicity analysis between control group and TiO_2_ nanoparticles synthesized by *Bacillus subtilis* at various days with SE (Standard Error).

Cell Viability of Control Group (Water)	Cell Viability of TiO_2_ Nanoparticles	Mean Difference (SE)	*p* Value
Cell viability of water at day 1	Cell viability of TiO_2_ nanoparticles at day 1	1.36 (0.34)	0.001
Cell viability of water at day 3	Cell viability of TiO_2_ nanoparticles at day 3	3.93 (0.34)	0.00
Cell viability of water at day 7	Cell viability of TiO_2_ nanoparticles at day 7	6.81 (0.34)	0.00
Cell viability of water at day 21	Cell viability of TiO_2_ nanoparticles at day 21	8.29 (0.34)	0.00
Cell viability of water at day 30	Cell viability of TiO_2_ nanoparticles day at 30	9.87 (0.34)	0.00

**Table 2:** Inter-group comparison of cytotoxicity analysis of TiO_2_ nanoparticles synthesized by *Bacillus subtilis* across days 1, 3, 7, 21 and 30.

Cell Viability of TiO_2_ Nanoparticles	Cell Viability Comparison at Different Days	Mean Difference (SE)	*p* Value
Cell viability of TiO_2_ nanoparticles at day 1	Cell viability of TiO_2_ nanoparticles at day 3	2.57 (0.34)	0.00
	Cell viability of TiO_2_ nanoparticles at day 7	5.45 (0.34)	0.00
	Cell viability of TiO_2_ nanoparticles at day 21	6.93 (0.34)	0.00
	Cell viability of TiO_2_ nanoparticles at day 30	8.51 (0.34)	0.00
Cell viability of TiO_2_ nanoparticles at day 3	Cell viability of TiO_2_ nanoparticles at day 1	−2.57 (0.34)	0.00
	Cell viability of TiO_2_ nanoparticles at day 7	2.88 (0.34)	0.00
	Cell viability of TiO_2_ nanoparticles at day 21	4.36 (0.34)	0.00
	Cell viability of TiO_2_ nanoparticles at day 30	5.94 (0.34)	0.00
Cell viability of TiO_2_ nanoparticles at day 7	Cell viability of TiO_2_ nanoparticles at day 1	−5.45 (0.34)	0.00
	Cell viability of TiO_2_ nanoparticles at day 3	−2.88 (0.34)	0.00
	Cell viability of TiO_2_ nanoparticles at day 21	1.48 (0.34)	0.00
	Cell viability of TiO_2_ nanoparticles at day 30	3.06 (0.34)	0.00
Cell viability of TiO_2_ nanoparticles at day 21	Cell viability of TiO_2_ nanoparticles at day 1	−6.93 (0.34)	0.00
	Cell viability of TiO_2_ nanoparticles at day 3	−4.36 (0.34)	0.00
	Cell viability of TiO_2_ nanoparticles at day 7	−1.48 (0.34)	0.00
	Cell viability of TiO_2_ nanoparticles at day 30	1.58 (0.34)	0.00
Cell viability of TiO_2_ nanoparticles at day 30	Cell viability of TiO_2_ nanoparticles at day 1	−8.51 (0.34)	0.00
	Cell viability of TiO_2_ nanoparticles at day 3	−5.94 (0.34)	0.00
	Cell viability of TiO_2_ nanoparticles at day 7	−3.06 (0.34)	0.00
	Cell viability of TiO_2_ nanoparticles at day 21	−1.58 (0.34)	0.00

**Table 3:** Inter-group comparisons of compressive strength of GIC restorative material containing various concentrations of TiO_2_ nanoparticles.

Different % of TiO_2_ Nanoparticles Incorporated in GIC Restorative Material (TiO_2_-GIC Samples)	Comparison Groups of TiO_2_-GIC Samples	Mean Difference	Standard Error (S.E)	*p* Value
0% TiO_2_-GIC Sample	3% TiO_2_-GIC Sample	−3.76	0.19	0.00
	5% TiO_2_-GIC Sample	−7.88	0.19	0.00
	7% TiO_2_-GIC Sample	−4.27	0.19	0.00
	10% TiO_2_-GIC Sample	−0.47	0.19	0.11
3% TiO_2_-GIC Sample	0% TiO_2_-GIC Sample	3.76	0.19	0.00
	5% TiO_2_-GIC Sample	−4.12	0.19	0.00
	7% TiO_2_-GIC Sample	−0.51	0.19	.066
	10% TiO_2_-GIC Sample	3.29	0.19	0.00
5% TiO_2_-GIC Sample	0% TiO_2_-GIC Sample	7.88	0.19	0.00
	3% TiO_2_-GIC Sample	4.12	0.19	0.00
	7% TiO_2_-GIC Sample	3.61	0.19	0.00
	10% TiO_2_-GIC Sample	7.41	0.19	0.00
7% TiO_2_-GIC Sample	0% TiO_2_-GIC Sample	4.27	0.19	0.00
	3% TiO_2_-GIC Sample	0.51	0.19	0.066
	5% TiO_2_-GIC Sample	−3.61	0.19	0.00
	10% TiO_2_-GIC Sample	3.80	0.19	0.00
10% TiO_2_-GIC Sample	0% TiO_2_-GIC Sample	0.47	0.19	0.11
	3% TiO_2_-GIC Sample	−3.29	0.19	0.00
	5% TiO_2_-GIC Sample	−7.41	0.19	0.00
	7% TiO_2_-GIC Sample	−3.80	0.19	0.00

**Table 4:** Inter-group comparisons of flexural strength of GIC restorative material containing various concentrations of TiO_2_ nanoparticles.

Different % of TiO_2_ Nanoparticles Incorporated in GIC Restorative Material (TiO_2_-GIC Samples)	Comparison Groups of TiO_2_-GIC Samples	Mean Difference	Standard Error (S.E)	*p*-Value
0% TiO_2_-GIC Sample	3% TiO_2_-GIC Sample	−5.30	0.14	0.00
	5% TiO_2_-GIC Sample	−10.28	0.14	0.00
	7% TiO_2_-GIC Sample	−6.79	0.14	0.00
	10% TiO_2_-GIC Sample	−1.00	0.14	0.00
3% TiO_2_-GIC Sample	0% TiO_2_-GIC Sample	5.30	0.14	0.00
	5% TiO_2_-GIC Sample	−4.98	0.14	0.00
	7% TiO_2_-GIC Sample	−1.49	0.14	0.00
	10% TiO_2_-GIC Sample	4.30	0.14	0.00
5% TiO_2_-GIC Sample	0% TiO_2_-GIC Sample	10.28	0.14	0.00
	3% TiO_2_-GIC Sample	4.98	0.14	0.00
	7% TiO_2_-GIC Sample	3.49	0.14	0.00
	10% TiO_2_-GIC Sample	9.28	0.14	0.00
7% TiO_2_-GIC Sample	0% TiO_2_-GIC Sample	6.79	0.14	0.00
	3% TiO_2_-GIC Sample	1.49	0.14	0.00
	5% TiO_2_-GIC Sample	−3.49	0.14	0.00
	10% TiO_2_-GIC Sample	5.79	0.14	0.00
10% TiO_2_-GIC Sample	0% TiO_2_-GIC Sample	1.00	0.14	0.00
	3% TiO_2_-GIC Sample	−4.30	0.14	0.00
	5% TiO_2_-GIC Sample	−9.28	0.14	0.00
	7% TiO_2_-GIC Sample	−5.79	0.14	0.00

## Data Availability

The data presented in this study will be available on request from the corresponding author.
