# Correction: Mansoor et al. Synthesis and Characterization of Titanium Oxide Nanoparticles with a Novel Biogenic Process for Dental Application. *Nanomaterials* 2022, *12*, 1078

**DOI:** 10.3390/nano14181485

**Published:** 2024-09-13

**Authors:** Afsheen Mansoor, Muhammad Talal Khan, Mazhar Mehmood, Zohaib Khurshid, Muhammad Ishtiaq Ali, Asif Jamal

**Affiliations:** 1 Department of Microbiology, Quaid-i-Azam University, Islamabad 45320, Pakistan; drafsheen@szabmu.edu.pk (A.M.); ishimrl@qau.edu.pk (M.I.A.); 2 Department of Dental Material Sciences, School of Dentistry, Shaheed Zulfiqar Ali Bhutto Medical University, Islamabad 44080, Pakistan; 3 Department of Dental Biomaterials, Bakhtawar Amin Medical and Dental College, Multan 60650, Pakistan; tkhaan747@gmail.com; 4 Department of Metallurgy and Materials Engineering, Pakistan Institute of Engineering and Applied Sciences, Islamabad 45650, Pakistan; mazhar@pieas.edu.pk; 5 Department of Prosthodontics and Dental Implantology, College of Dentistry, King Faisal University, Al-Hofuf 31982, Saudi Arabia; zsultan@kfu.edu.sa

## Text Correction

In the original publication [1], the data for the Atomic Force Microscopy (AFM) analysis were missing. A correction has been made to Section 2.2. Characterization of TiO_2_.

“A tapping-mode AFM probe with a cantilever tip, HQ: NSC-16 (Mikromasch), was used.”

## Error in Figure

In the original publication [1], there was a mistake in Figure 9 as published. Figure 9A–F were erroneously included from the same raw data source. The corrected Figure 9A–F appear below.

The authors state that the scientific conclusions are not affected because of this correction. This correction was approved by the Academic Editor. The original publication has also been updated.

## Figures and Tables

**Figure 9:**
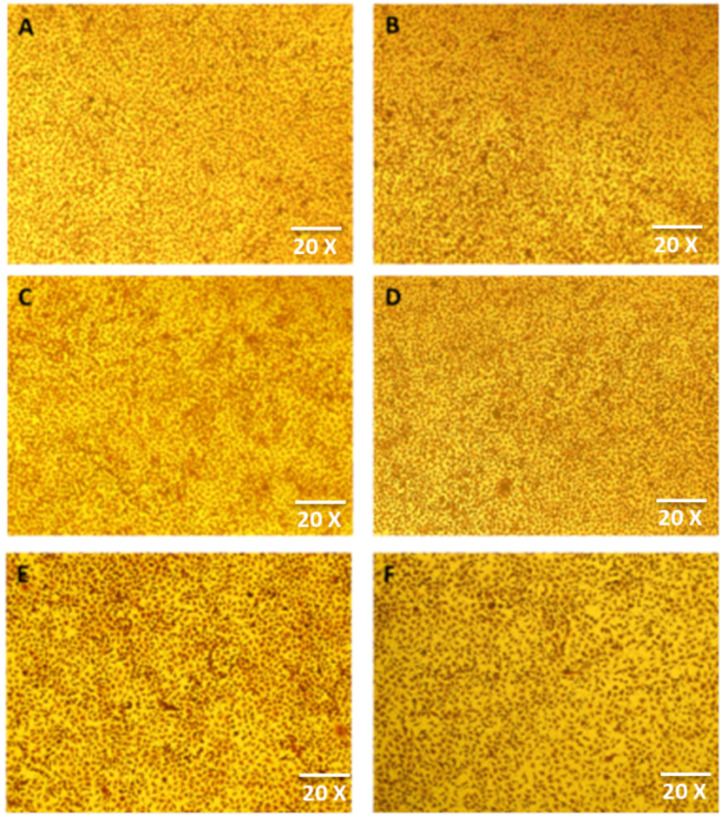
Morphology of (**A**) cells in control group at day 1, (**B**) TiO_2_ nanoparticle-treated cells at day 1, (**C**) cells in control group at day 7, (**D**) TiO_2_ nanoparticle-treated cells at day 7, (**E**) cells in control group at day 30, and (**F**) TiO_2_ nanoparticle-treated cells at day 30.
